# Missed opportunities for concomitant HPV vaccination among childhood cancer survivors

**DOI:** 10.1002/cam4.4492

**Published:** 2022-01-14

**Authors:** Joemy M. Ramsay, Heydon K. Kaddas, Judy Y. Ou, Deanna Kepka, Anne C. Kirchhoff

**Affiliations:** ^1^ Cancer Control and Population Sciences Huntsman Cancer Institute Salt Lake City Utah USA; ^2^ College of Nursing University of Utah Salt Lake City Utah USA; ^3^ Department of Pediatrics University of Utah Salt Lake City Utah USA

**Keywords:** childhood cancer survivors, HPV, immunizations, missed opportunities

## Abstract

**Purpose:**

Childhood cancer survivors are at higher risk of human papillomavirus (HPV)‐related second cancers than adolescents without cancer, yet their HPV vaccination uptake is lower. Using a statewide sample, we evaluated whether survivors are at higher risk of missed opportunities for concomitant HPV vaccination.

**Methods:**

From statewide healthcare data, we identified encounters where vaccines were received. Concomitant HPV vaccine missed opportunities were defined as a vaccine encounter where the HPV vaccine was not administered, although eligibility criteria were met. From these encounters, our sample included 327 survivors identified from the Utah Cancer Registry, diagnosed 2000–2016 at ages 0–9, and a birth year and sex‐matched sample without cancer from the general population (*n* = 1,911). Mixed‐effects Poisson regression estimated the rate of concomitant missed opportunities per vaccine encounter and 95% confidence intervals by vaccine encounter type (all vaccines, flu shot only, or adolescent/catch‐up) from 2013 to 2016.

**Results:**

Survivors had more concomitant HPV vaccine missed opportunities than the population sample (70.0% vs. 59.0%). On average, survivors were 12% more likely to have missed opportunities at vaccine encounters and 4% more likely at flu shot only encounters. The predicted excess risk of concomitant missed opportunities for survivors ranged from 0.5 per10 vaccine encounters to 1.1 per10 vaccine encounters. Higher parental education, rurality, younger first vaccine age, and chemotherapy were associated with missed opportunities.

**Conclusions:**

Childhood cancer survivors have more missed opportunities for concomitant HPV vaccination than a population sample. As flu shots should be administered annually, providers have a regular opportunity to recommend and deliver the HPV vaccine to survivors.

## Abstract 2

Childhood cancer survivors face a higher risk of HPV‐related cancers than adolescents without a cancer history; however, their vaccination rates tend to be lower. We assessed HPV vaccine missed opportunities among age‐eligible childhood cancer survivors and found significantly more missed opportunities for survivors than adolescents without a cancer history.

## 1 INTRODUCTION

Childhood cancer survivors face increased risk for developing subsequent neoplasms, including human papillomavirus (HPV)‐related cancers, compared to individuals without a cancer history.^1^, ^2^ Cancer treatments can cause prolonged immunosuppression, increasing the likelihood of persistent HPV infection, and higher risk of HPV‐related malignancies.^1^, ^3^, ^4^, ^5^ In spite of childhood cancer survivors’ increased risk, their HPV vaccination rates remain low, with one study reporting series completion rates of only 13.5% for survivors compared to 20.8% for cancer‐free individuals.^6^

The Advisory Committee on Immunization Practices (ACIP) recommends adolescents aged 11–12 years receive the following vaccinations: HPV, first dose of meningococcal conjugate (MenACWY), tetanus, and reduced diphtheria toxoids and acellular pertussis (Tdap), and annual flu shot.^7^, ^8^, ^9^, ^10^ Despite the shown efficacy and safety of the HPV vaccine only 54% of adolescents in the United States (US) were up to date with HPV vaccine recommendations in 2019. HPV vaccination rates are also low compared to rates for MenACWY (89%) and Tdap (90%),^7^ indicating that adolescents are missing opportunities for HPV vaccination at other adolescent vaccination encounters. Survivors’ risk for concomitant missed opportunities, healthcare encounters where they receive another vaccine but not the HPV vaccine, has not been assessed.

Childhood cancer survivors may be at increased risk of HPV vaccine missed opportunities for many reasons. The most important factor prompting HPV vaccination, for children with and without a cancer history, is a strong provider recommendation.^6^, ^11^, ^12^, ^13^, ^14^, ^15^, ^16^, ^17^ However, earlier studies show that survivors are less likely to receive a provider recommendation for the HPV vaccine than adolescents without a cancer history.^6^, ^17^ Additionally, HPV vaccinations are typically administered in primary care settings,^18^ posing an additional barrier as up to 70% of primary care providers (PCPs) lack confidence in providing immunizations to survivors even though ≥80% will encounter survivors in their practice.^19^, ^20^ However, it is unclear whether these barriers affect HPV vaccination rates more than other adolescent vaccines and place survivors at higher risk for concomitant missed opportunities.

Understanding concomitant missed opportunities among survivors is important for identifying strategies to improve HPV vaccine uptake in this vulnerable population. We report on demographic and clinical predictors of concomitant HPV vaccine missed opportunities among a statewide cohort of Utah‐based children and adolescents diagnosed with childhood cancer. Drawing on electronic health records (EHRs) from two major health care systems, a statewide insurance claims database, and data from an immunization information system, we compared concomitant HPV vaccine missed opportunities for survivors and a cancer‐free birth year and sex‐matched sample drawn from the general population.

## 2 METHODS

All study procedures and materials were approved by the University of Utah institutional review board (IRB).

### 2.1 Data sources and sample

#### 2.1.1 Datasets

Study data are from the Utah Population Database (UPDB), a powerful statewide population registry that contains linked demographic, residential, clinical, and vital status records for over eight million individuals.^21^, ^22^ UPDB also links with the Utah Cancer Registry (UCR), a Surveillance, Epidemiology, and End Results (SEER) program registry, and healthcare encounter data from the two major healthcare systems in Utah (Intermountain Healthcare (IHC) and University of Utah Healthcare Systems (UUHC)). IHC & UUHC account for ~80% of medical encounters in the state and both maintain data warehouses that record diagnoses and clinical histories for all patients.^21^ Through UPDB, we also accessed records from the Utah Statewise Immunization System (USIIS) and Utah's All Payer Claims Database (APCD).^23^ APCD contains claims for Medicaid and all commercial insurance carriers licensed in Utah covering ≥2500 Utahns.

#### 2.1.2 Sample and eligibility

Sample eligibility was based on the years of APCD availability (2013–2016), as APCD provided the majority of vaccination data. We identified individuals diagnosed with childhood cancer from UCR records. Eligible survivors were diagnosed with cancer defined by an International Classification of Childhood Cancer (ICCC) code, excluding non‐malignant and in situ cancers, ≤9 years of age from 2000 to 2016.^24^ UPDB identified a population‐based sample without cancer. The population sample was selected from Utah birth certificates with three members of the population sample matched to each survivor on birth year and sex.

**FIGURE 1:**
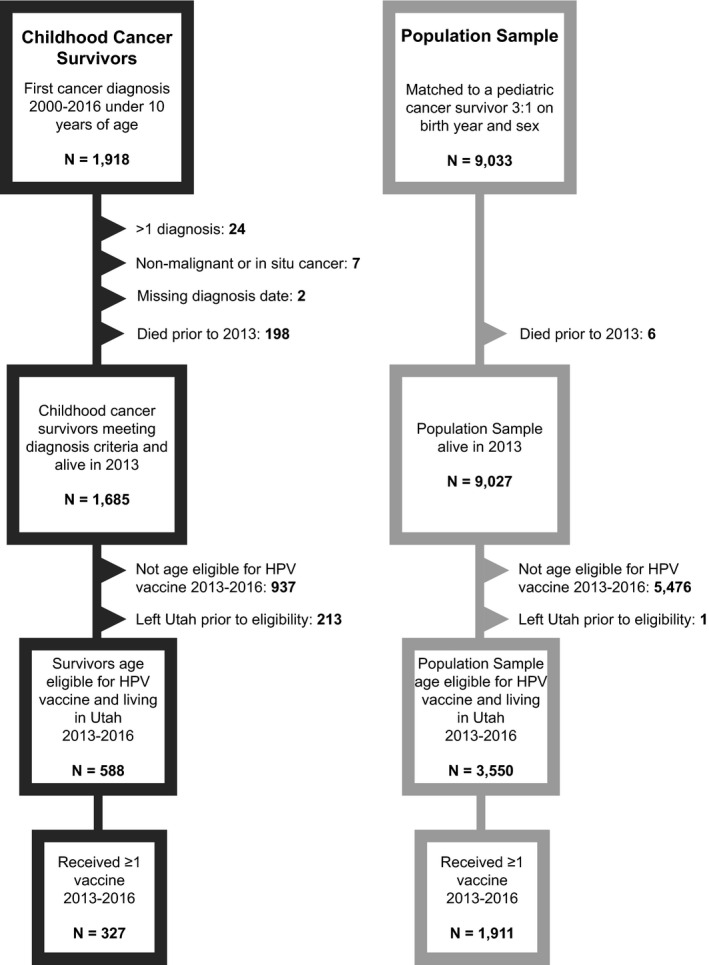
Criteria for inclusion in analysis of HPV vaccine missed opportunities

We included survivors and members of the population sample in this analysis who were living in Utah in 2013 and were age eligible for the HPV vaccine based on the ACIP and Centers for Disease Control and Prevention (CDC) guidelines during 2013–2016.^8^, ^9^, ^10^ As ACIP and CDC recommend HPV vaccination at 11–12 years of age, with vaccination as early as 9 years of age, participants needed to be 9–13 years old in 2013–2016.^8^, ^9^, ^10^ Utah residency was determined through UPDB or by zip code from driver license and voter registration records or healthcare encounters. A total of 1900 individuals, 261 survivors and 1639 of the population sample, did not receive any vaccines during the study window and were excluded from the concomitant missed opportunity analysis, leaving 327 survivors and 1911 population sample who received ≥1 vaccine (Figure 1). Demographic and clinical characteristics for all individuals who met the eligibility criteria for this analysis are summarized in Table S1 by vaccination status within the study window.

### 2.2 Measures

#### 2.2.1 Vaccination encounters

We identified vaccination encounters from claims and encounter records (IHC, UUHC, APCD, and USIIS) using Current Procedural Terminology (CPT) codes and National Drug Codes (NDCs). Text string matching was used to identify vaccines in claims missing NDCs. CPTs and NDCs were mapped to the vaccine administered code list (CVX) from the CDC.^25^ CVX includes all vaccines ever available in the United States and maps individual CVX codes to groups containing vaccines that either (a) all vaccinate against a specific disease and fulfill ACIP schedule requirements or (b) vaccinate against more than one disease but are clinically grouped.^26^

We classified each vaccination encounter by vaccine(s) administered. Visits were grouped as: (1) only HPV vaccine, (2) only flu shot, (3) adolescent/catch‐up vaccines, or (4) other vaccine(s). Adolescent/catch‐up vaccines were defined as vaccines included in the standard adolescent schedule (HPV, MenACWY, Tdap, and flu) or the recommended catch‐up schedule for adolescents (Hepatitis A and B, polio, MMR, and varicella). “Other” vaccines included vaccinations not included in adolescent/catch‐up schedules (HIB, pneumococcal, typhoid, and zoster). We excluded vaccines used to test for or treat disease (rabies, varicella zoster immune globulin, and tuberculin skin tests). None of the vaccines of interest allow for dosing intervals of less than 1 month. We therefore considered vaccines in the same CVX group that took place within 21 days of each other as belonging to the same encounter as the minor variation in timing likely represents an artifact of the different data collection processes within the various data and healthcare systems.

#### 2.2.2 Missed opportunities for concomitant HPV vaccination

We defined missed opportunities for an HPV vaccine dose as a healthcare encounter where the individual was eligible for the HPV vaccine and received ≥1 vaccination(s) without concomitant HPV vaccination. We did not count encounters as missed opportunities if a dose of the HPV vaccine was received or if other vaccines were received within the minimum dosing intervals for subsequent HPV vaccination: 4 weeks between the first and second dose and 12 weeks between the second and third dose. Vaccination encounters without concomitant HPV vaccination that took place after the minimum HPV vaccine dosing intervals had passed were considered missed opportunities, even if the individual later went on to complete the series. Consistent with ACIP and CDC recommendations for 2013–2016, HPV vaccination was considered complete if the three‐dose series was finished within 18 months.

#### 2.2.3 Other measures

We generated variables for race/ethnicity, parental education, sex, and birthdate using UPDB, UUHC, IHC, APCD, and UCR records. Race/ethnicity was classified as either non‐Hispanic White or Hispanic/other. Average parental education was based on educational attainment listed for each parent on the subject's birth certificate from UPDB. Cancer‐specific measures were obtained through UCR, including age at diagnosis, diagnosis date, and whether or not they received chemotherapy and/or radiotherapy as treatment for their cancer. We grouped ICCC diagnoses into the following categories: leukemia, lymphoma, central nervous system (CNS) neoplasms, solid tumors (neuroblastoma, retinoblastoma, renal tumors, hepatic tumors, and germ cell tumors), sarcomas/bone tumors, and epithelial neoplasms.

### 2.3 Follow‐up

For survivors, eligibility for concomitant missed opportunities began on: their 9th birthday, their cancer diagnosis date, or January 1, 2013, whichever was latest. Individuals in the population sample were considered eligible for missed opportunities for concomitant HPV vaccine missed opportunities on their 9th birthday or January 1, 2013, whichever was latest. Follow‐up for survivors and the population sample started at their first vaccine encounter following eligibility and was bounded by the dates APCD was available: January 1, 2013–December 31, 2016. Participants were considered censored if they died, as confirmed through a death certificate, or if they left Utah. Follow‐up ended if the three‐dose HPV vaccination series was completed. Matched survivors and members of the population sample who met eligibility criteria but did not receive vaccinations during the study period were excluded from the analysis.

### 2.4 Statistical analyses

Demographic characteristics were compared between survivors and the population sample using chi‐squared and *t*‐test statistics. We evaluated concomitant HPV vaccine missed opportunities for survivors and the population sample using count of missed opportunities for the duration of the follow‐up period. Analyses were conducted for all vaccination encounters combined and stratified by the type of vaccine encounter: adolescent/catch‐up or flu shot only. There were insufficient vaccine encounters classified as “Other” for assessment in stratified models. Missed opportunities were also examined by whether or not individuals had ever received an HPV vaccine.

We calculated the rate of concomitant HPV vaccine missed opportunities. To do this, we used mixed‐effects Poisson regression with robust standard errors and exchangeable covariance structure. This modeling approach was selected to decrease the likelihood of biased estimates due to any potential clustering between survivors and their sex‐ and birth year‐matched population sample. We also calculated the expected excess number of concomitant HPV vaccine missed opportunities by subtracting the estimated count of missed opportunities for the population sample from that of survivors. Stratified models were used to assess associations between demographic characteristics and missed opportunities. We used multivariable Poisson regression with robust standard errors for survivor‐only models to examine demographic, treatment, and clinical risk factors for concomitant HPV vaccine missed opportunities. The total number of vaccine encounters was used as the offset in all Poisson models. All models were adjusted for sex, race/ethnicity, and age at first vaccination in the study period with *α* = 0.05 used for all statistical tests. Statistical analyses were performed in Stata version 14.2.

## 3 RESULTS

Demographic and clinical characteristics of survivors and the population sample who received ≥1 vaccine during the study are summarized in Table 1. Compared to the population sample, survivors were more likely to be other race/ethnicity, have private health insurance, and to be younger when they received their first vaccine within the study period and entered the cohort. Seven survivors (2.1%) died during follow‐up, with no deaths in the population sample.

**TABLE 1:** Demographic and clinical characteristics of childhood cancer survivors and the population sample who received ≥1 vaccination during the study window (*N* = 2238)

	Survivors		Population sample		*p*‐value^a^
	*N* = 327		*N* = 1911		
	Mean (SD)	Range	Mean (SD)	Range	
Age at cohort entry (first vaccination)	10.6 (2)	9–16	11.2 (2)	9–16	**<0.001**
	*n*	%	*n*	%	
Sex					
Female	144	44.0	910	47.6	0.230
Male	183	56.0	1001	52.4	
Race/ethnicity					
Non‐Hispanic White	253	77.4	1569	82.1	**0.042**
Other race/ethnicity	74	22.6	342	17.9	
Parental education^b^					
<High school	49	15.0	259	13.6	0.392
High school/GED	90	27.5	586	30.7	
Some college/AA	88	26.9	547	28.6	
≥College	66	20.2	493	25.8	
Rural/urban					
Urban	305	93.3	1788	93.6	0.843
Ever rural	22	6.7	123	6.4	
Insurance at first vaccine in study window					
Uninsured/no record of insurance	≤10		17	0.9	**0.005**
Public	55	16.8	338	17.7	
Private	262	80.1	1429	74.8	
Other	≤10		127	6.6	
Age at HPV series initiation^c^					
9–10	≤10		22	1.2	0.561
11–12	98	30.0	690	36.1	
13–16	27	8.3	210	11.0	
ICCC diagnosis group					
Leukemia	113	34.6			
Lymphoma	26	8.0			
CNS	70	21.4			
Solid tumors	83	25.4			
Sarcomas/bone	26	8.0			
Epithelial	≤10				
Treatment^d^					
Chemotherapy	239	73.1			
Radiation	75	22.9			
Age at diagnosis					
0–4	192	58.7			
5–9	135	41.3			
Year of diagnosis					
2000–2004	61	18.7			
2005–2010	179	54.7			
2011–2016	87	26.6			

Note

Percentages are suppressed when count is ≤10.

^a^ Chi‐squared or Fisher's exact *p*‐value: survivors versus population sample; *p* < 0.05 bolded.

^b^ Missing: 34 survivors and 26 population sample.

^c^ Total does not add up to 100%––only for individuals who received ≥1 HPV vaccine dose.

^d^ Chemotherapy: 1 missing, radiation: 1 missing.

Survivors were followed for an average of 1.91 years (SD = 1.18 years) with a mean of 3.7 vaccines received (range: 1–15) and the population sample was followed for an average of 2.10 years (SD = 1.17) with 3.5 vaccines received (range: 1–17, not shown). A total of 48.2% (*n* = 922) of the population sample and 39.8% (*n* = 130) of the survivors received ≥1 HPV vaccine (*p* = 0.004), with 10.2% (*n* = 195) of the population sample completing the three‐dose HPV series compared to 7.3% (*n* = 24) of the survivors (*p* = 0.107). Survivors and the population sample contributed a total of 5108 vaccine encounters during follow‐up (Table 2). Most vaccine encounters (60.8%) were missed opportunities for concomitant HPV vaccination, with more missed opportunities for survivors than members of the population sample, 70.0% versus 59.0% (*p* < 0.001, not shown). The expected number of missed opportunities was significantly higher for survivors than the population sample for all vaccination encounters and flu shot encounters, regardless of whether any HPV vaccine doses were ever received (Table 3). More missed opportunities among survivors were expected for flu shot encounters than adolescent/catch‐up vaccination encounters.

**TABLE 2:** Vaccine encounters during study for childhood cancer survivors and the population sample (*N* = 5108 vaccine encounters)

	Survivors *N* = 811 encounters		Population sample *N* = 4297 encounters	
	N	%	N	%
Adolescent/catch‐up^a^	245	30.2	1525	35.5
HPV vaccine only	110	13.6	846	19.7
Flu shot only	420	51.8	1711	39.8
Flu shot and HPV vaccine	30	3.7	203	4.7
Other^b^	≤10		12	0.3

Note

Percentages are suppressed when count is ≤10.

^a^ Includes HPV vaccine if it was administered as part of encounter.

^b^ Vaccines not considered catch‐up or normal adolescent.

**TABLE 3:** Excess number of expected concomitant HPV vaccine missed opportunities for childhood cancer survivors compared to the population sample by type of vaccine encounter^a^

	Any vaccine encounter		Type of vaccination			
			Adolescent/catch‐up		Flu shot	
	Rate of missed opportunities (95% CI)	Excess missed opportunities (95% CI)	Rate of missed opportunities (95% CI)	Excess missed opportunities (95% CI)	Rate of missed opportunities (95% CI)	Excess missed opportunities (95% CI)
Full cohort						
Survivors (*N* = 327)	7.0 (6.6–7.4)	**1.1 (0.6–1.5)**	4.1 (3.5–4.7)	0.5 (−0.2–1.2)	9.3 (9.0–9.5)	**0.5 (0.2–0.8)**
Population sample (*N* = 1911)	5.9 (5.7–6.1)		3.6 (3.4–3.9)		8.8 (8.7–9.0)	
No HPV vaccines received						
Survivors (*N* = 197)	5.0 (4.3–5.6)	**0.8 (0.1–1.5)**	3.4 (2.7–4.1)	0.2 (−0.6–0.9)	8.1 (7.4–8.7)	**1.0 (0.3–1.8)**
Population sample (*N* = 989)	4.2 (3.9–4.4)		3.2 (2.9–3.5)		7.1 (6.7–7.4)	
≥1 HPV vaccine received						
Survivors (*N* = 94)	4.3 (3.9–4.8)	**0.9 (0.4–1.4)**	1.5 (1.0–1.9)	0.3 (−0.2–0.8)	8.3 (7.7–8.9)	**0.7 (0.1–1.3)**
Population sample (*N* = 493)	3.4 (3.2–3.6)		1.2 (1.0–1.3)		7.6 (7.3–7.9)	

^a^ Models adjusted for sex; reported as rate of missed opportunities per 10 vaccine encounters; reported as expected number of excess missed opportunities per 10 vaccine encounters for survivors relative to population sample; *p* < 0.05 bolded.

Survivors had significantly higher rates of concomitant HPV vaccine missed opportunities than the population sample for all vaccine encounters and flu shot encounters specifically (Table 4). Relative to members of the population sample in the same demographic subgroup, significantly higher rates of missed opportunities per vaccine encounter were observed for males, non‐Hispanic Whites, urban residents, and survivors who were other race/ethnicity, whose parents attained a high school or associates degree, those who were publicly or privately insured, or who were 9–10 years old at their first vaccine in the study window. Increased rates of missed opportunities for concomitant HPV vaccination were also seen during adolescent/catch‐up and flu shot vaccination encounters for survivors residing in urban areas, whose parents had a high school education, and who received their first vaccine in the study window when they were 9–10 years of age. Rates of flu shot missed opportunities were significantly higher for survivors than population controls for adolescents who were female, non‐Hispanic White, whose parents had at least a college degree, were 11–12 years old at first vaccination in the study, and were privately insured.

**TABLE 4:** Incidence rate ratios (IRRs) and 95% CIs for concomitant HPV vaccine missed opportunities by vaccine encounter type stratified on demographic characteristics for childhood cancer survivors compared to the population sample^a^

	Any vaccine encounter		Adolescent/catch‐up encounters		Flu shot encounters	
	IRR	95% CI	IRR	95% CI	IRR	95% CI
Full sample	**1.12**	**1.06–1.20**	1.03	0.88–1.22	**1.04**	**1.01–1.08**
Sex						
Female	1.10	0.99–1.21	1.18	0.90–1.55	**1.05**	**1.01–1.10**
Male	**1.16**	**1.07–1.26**	1.21	1.00–1.48	1.04	1.00–1.08
Race/ethnicity						
Other race/ethnicity	**1.21**	**1.03–1.42**	1.46	0.96–2.21	1.05	0.96–1.15
Non‐Hispanic White	**1.11**	**1.04–1.19**	1.16	0.97–1.38	**1.04**	**1.01–1.07**
Parental education						
<High school	1.03	0.83‐–1.27	0.95	0.58–1.56	0.97	0.85–1.11
High school/GED	**1.18**	**1.05–1.33**	**1.38**	**1.03–1.84**	**1.08**	**1.03–1.13**
Some college/AA	**1.16**	**1.04–1.29**	1.15	0.87–1.51	1.04	0.98–1.09
≥College	1.10	0.98–1.24	1.18	0.81–1.70	**1.06**	**1.01–1.11**
Rural/urban						
Urban	**1.12**	**1.05–1.20**	**1.19**	**1.00–1.41**	**1.04**	**1.01–1.08**
Ever rural	1.15	0.93–1.43	1.13	0.54–2.38	1.03	0.95–1.12
Insurance						
No record of insurance	1.16	0.59–2.27	—	—	1.48	0.80–2.71
Public	**1.22**	**1.03–1.44**	1.33	0.87–2.04	1.05	0.97–1.14
Private	**1.11**	**1.04–1.19**	1.18	0.99–1.42	**1.04**	**1.01–1.08**
Other	1.12	0.86–1.47	0.81	0.25–2.65	1.04	0.87–1.25
Age at first vaccine						
9–10	**1.09**	**1.02–1.17**	**1.34**	**1.03–1.74**	1.02	0.99–1.05
11–12	1.13	0.99–1.29	1.04	0.83–1.30	**1.09**	**1.02–1.17**
13–16	1.14	0.92–1.40	1.14	0.76–1.71	1.00	0.88–1.14

^a^ Models adjusted for sex, race/ethnicity, and age at first vaccine; cells left blank when there were insufficient events for estimation; *p* < 0.05 bolded.

Among survivors, there were few significant differences in the rate of missed opportunities for concomitant HPV vaccination by demographic and clinical characteristics (Table S2). Across all vaccine encounters and for both encounter types, missed opportunities occurred at significantly higher rates for survivors who were treated with chemotherapy (IRR = 1.35, 95% CI: 1.12–1.62), and tended to increase with increasing parental education (trend *p*‐value <0.001). Missed opportunities declined with increasing age at first vaccine in the study window for all vaccine encounters (trend *p*‐value <0.001) and flu shot encounters (trend *p*‐value = 0.012). Survivors who lived in rural areas (IRR = 1.27, 95% CI: 1.09–1.49) and were diagnosed at older ages (per year: IRR = 1.04, 95% CI: 1.02–1.06) also had significantly higher rates of missed opportunities. For flu shot encounters, a significant decrease in the rate of missed opportunities was observed for survivors who were treated with radiation (IRR = 0.92, 95% CI: 0.85–0.99).

## 4 DISCUSSION

The Children's Oncology Group recommends routine administration of the HPV vaccine for all age‐eligible childhood cancer survivors,^8^, ^10^, ^27^, ^28^, ^29^ however, HPV vaccine uptake remains low for this high‐risk population.^6^, ^15^, ^16^, ^17^ In our statewide sample, we found that childhood cancer survivors are at significantly higher risk of experiencing missed opportunities for concomitantly receiving the HPV vaccine than adolescents without a cancer history. In survivor‐only analyses, we observed that survivors who lived in rural areas, were younger when they received their first vaccine, or whose parents had higher average educational attainment were more likely to have missed opportunities for concomitant HPV vaccination in the study period. Treatment with chemotherapy was also associated with a greater number of missed opportunities.

Prior studies have shown that a strong provider recommendation is one of the most important motivating factors for HPV vaccination.^6^, ^11^, ^12^, ^13^, ^14^, ^15^, ^16^, ^17^ The majority of concomitant HPV vaccine missed opportunities occurred at annual flu shot encounters. As most adolescents receive the flu shot at either a doctor's office, clinic, hospital, or some other medical facility, flu shot encounters provide a viable avenue for providers to take advantage of their limited contact with adolescent survivors and improve HPV vaccine uptake.^11^, ^30^ Also, as prior work from our group has identified that an oncologist or PCP recommendation is critical to vaccine decision‐making for survivors and caregivers, this could be a potential explanation for the higher rate of concomitant missed opportunities among survivors.^31^ PCPs report a lack familiarity with follow‐up care guidelines, including post‐treatment vaccination guidance.^19^, ^20^ Thus, for survivors who have transitioned back to primary care, increased coordination is needed between PCPs and oncologists.^18^ While we were unable to investigate provider recommendations in this study, future work on HPV vaccination among cancer survivors will require better understanding of provider practices.

Survivors treated with chemotherapy had more missed opportunities for concomitant HPV vaccination than survivors who did not receive chemotherapy, indicating that providers may need guidance regarding vaccine safety following cancer treatments. At the same time, caregivers of survivors have cited lack of information on the vaccine and concerns over potential side effects as their primary reason for not vaccinating their child.^32^ The HPV vaccine is indicated for immunocompromised individuals and the Children's Oncology Group (COG) recommends resuming vaccines 3–6 months post‐therapy and 6–12 months for live vaccines, suggesting that education for providers and caregivers may be necessary for increasing HPV vaccination uptake for survivors.^27^, ^33^ Most survivors also show protective titers following vaccination indicating that post‐treatment vaccination is effective.^33^ This is especially important as survivors who received more intensive treatments, including chemotherapy, have higher risk for long‐term immunosuppression, increasing their risk for persistent HPV infection and HPV‐related cancers.^4^

Among survivors, trends in increasing rates of missed opportunities for concomitant HPV vaccination were observed by rurality, age at first adolescent vaccine, parental education, and diagnosis age. Rural residents tend to have higher incidence of HPV‐related cancers and lower rates of HPV vaccination due to a complex mixture of interpersonal, organizational, and community/societal factors, including a lower availability of cancer prevention and treatment services than individuals living in urban areas.^14^, ^34^ The higher rates of missed opportunities seen for both rural survivors and rural member fo the population sample, with no significant differences between the two, are reflective of the larger need to increase rural HPV vaccination rates and indicates that additional efforts and interventions should be focused on motivating HPV vaccination during vaccination encounters for rural individuals. Additionally, Utah is a highly religious state, particularly in rural communities which may further influence vaccine beliefs and parental behaviors.^35^ In addition to rurality, earlier studies have shown that higher parental education is associated with a lower parental probability of HPV vaccination initiation and intent.^36^, ^37^ In non‐cancer populations, this trend is speculated to be due to a myriad of reasons, including less experience with HPV‐related diseases and greater access to healthcare among higher income families, however, how the phenomena plays out among childhood cancer survivors is unknown and is an important area for future research.

We also observed increased missed opportunities with older age at diagnosis, indicating that a childhood cancer diagnosis closer to adolescence can be highly disruptive to receiving the HPV vaccination. Parents could feel concerned with having their child receive multiple vaccinations so soon after treatment and the HPV vaccine is the most likely to be delayed during adolescence.^38^ Existing HPV vaccination studies focused on childhood cancer survivors also show that survivors tend to be vaccinated against HPV later in adolescence, this is consistent with the higher rates of concomitant HPV vaccine missed opportunities observed for survivors in our cohort who were younger, 9–10 years, when they received their first vaccine in the study window.^6^, ^16^, ^17^ The HPV vaccine is most effective when it is administered to younger adolescents, before sexual activity and potential exposure to HPV.^6^, ^15^, ^16^, ^17^ Evidence also suggests that children who initiate the vaccine at earlier ages, 9–10 years, are much more likely to complete the series.^39^Recommendations for survivors are to receive the full three dose series of the HPV vaccine, an earlier initiation age may therefore provide an important avenue towards maximize the efficacy of the HPV vaccine and increase the likelihood of completion. At a minimum, further efforts should be made to increase HPV vaccination rates at the CDC recommendation age of 11–12 although the American Academy of Pediatrics has begun to recommend routine HPV vaccination beginning at 9 years in order to offer more flexibility in introducing the vaccine and the ability to disentangle the HPV vaccine from conversations around sexual activity.^38^

This study has certain limitations. Our study was conducted over a narrow time window, 2013–2016, due to limited availability of APCD data. Additionally, we defined missed opportunities for HPV vaccination as healthcare encounters where a vaccine was received without concomitant administration of the HPV vaccine. Therefore, this analysis did not capture primary care‐based missed opportunities among survivors and members of the population sample who did not receive any vaccinations during the study window. However, because survivors often have delayed transitions from oncology to primary care or could be more likely to experience conditions that contraindicate vaccination such as fevers, which are poorly documented in EHRs assessing primary care‐based missed opportunities are unlikely to provide an accurate picture of vaccination behaviors in childhood cancer survivors.

## 5 CONCLUSION

Although survivors of childhood cancer face greater risk for HPV‐related second cancers, our analysis showed they have significantly higher rates of missed opportunities for concomitant HPV vaccination than a population sample of adolescents without cancer, particularly for flu shot vaccine encounters. As the flu shot should be administered annually, providers have a regular opportunity to strongly recommend and deliver the HPV vaccine to survivors. Providers should emphasize the safety and efficacy of the vaccine and motivate vaccination against HPV within the recommended age range, particularly if survivors were treated with chemotherapy. Improved communication and coordination between oncology and primary care teams could also help to increase PCP comfort and knowledge surrounding follow‐up care for childhood cancer survivors.

## ETHICS STATEMENT

All study procedures and materials were approved by the University of Utah IRB.

## CONFLICT OF INTEREST

Dr. Kepka receives a small portion of her salary from a grant that is provided and supported by the American Cancer Society, who received funding from Merck, for the purpose of the “Mission: HPV Cancer Free Quality Improvement Initiative.”

The other authors have no conflicts of interest to disclose.

## AUTHOR CONTRIBUTIONS

Joemy Ramsay: Conceptualization, data curation, formal analysis, methodology, writing, and review/editing. Heydon Kaddas: Data curation, methodology, and review/editing. Judy Ou: Methodology and review/editing. Deanna Kepka: Conceptualization, funding acquisition, review/editing, and supervision. Anne Kirchhoff: Conceptualization, funding acquisition, methodology, project administration, resources, review/editing, and supervision.

## Supporting information

Table S1‐S2

## Data Availability

Research data are not shared.
