# Supplementary material for: Missed opportunities for concomitant HPV vaccination among childhood cancer survivors
Source: Cancer Med. 2022 Jan 14;11(4):1181–91. doi: 10.1002/cam4.4492 (PMC8855920; doi:10.1002/cam4.4492)
Supplement: Supplementary file 1 — Table S1‐S2 [file CAM4-11-1181-s001.docx]

| Supplemental Table 1: Demographic Characteristics of Childhood Cancer Survivors and the Population Sample by Vaccination Status during the Study Window (N=4,138) | | | | | | | | | | | | |
| --- | --- | --- | --- | --- | --- | --- | --- | --- | --- | --- | --- | --- |
|  |  | Survivors | | | | |  | Population Sample | | | | |
|  |  | N=588 | | | | |  | N=3,550 | | | | |
|  |  | No Vaccines | | ≥1 Vaccination | |  |  | No Vaccines | | ≥1 Vaccination | | p=0.422 |
|  |  | N=261 | | N=327 | |  |  | N=1,639 | | N=1,911 | |  |
|  |  | Mean (SD) | Range | Mean (SD) | Range | p-value ^a^ |  | Mean (SD) | Range | Mean (SD) | Range | p-value ^a^ |
|  | Entry Age (years) | 9.6 (1) | 9-15 | 10.6 (2) | 9-16 | **<0.001** |  | 9.7 (1) | 9-12 | 11.2 (2) | 9-16 | **<0.001** |
|  |  |  |  |  |  |  |  |  |  |  |  |  |
|  |  | n | % | n | % |  |  | n | % | n | % |  |
| Sex | |  |  |  |  | 0.931 |  |  |  |  |  | 0.479 |
|  | Female | 114 | 43.7 | 144 | 44.0 |  |  | 761 | 46.4 | 910 | 47.6 |  |
|  | Male | 147 | 56.3 | 183 | 56.0 |  |  | 878 | 53.6 | 1,001 | 52.4 |  |
| Race/Ethnicity | |  |  |  |  | 0.208 |  |  |  |  |  | 0.270 |
|  | Other Race/Ethnicity | 48 | 18.4 | 74 | 22.6 |  |  | 317 | 19.3 | 342 | 17.9 |  |
|  | Non-Hispanic White | 213 | 81.6 | 253 | 77.4 |  |  | 1,322 | 80.7 | 1,569 | 82.1 |  |
| Parental Education | |  |  |  |  | 0.677 |  |  |  |  |  | **<0.001** |
|  | <High School | 28 | 10.7 | 49 | 15.0 |  |  | 255 | 15.6 | 259 | 13.6 |  |
|  | High School/GED | 63 | 24.1 | 90 | 27.5 |  |  | 583 | 35.6 | 586 | 30.7 |  |
|  | Some College/AA | 71 | 27.2 | 88 | 26.9 |  |  | 429 | 26.2 | 547 | 28.6 |  |
|  | ≥College | 49 | 18.8 | 66 | 20.2 |  |  | 351 | 21.4 | 493 | 25.8 |  |
|  | Missing | 50 | 19.2 | 34 | 10.4 |  |  | 21 | 1.3 | 26 | 1.4 |  |
| Rural/Urban | |  |  |  |  | 0.195 |  |  |  |  |  | **0.001** |
|  | Urban | 234 | 89.7 | 305 | 93.3 |  |  | 1,483 | 90.5 | 1,788 | 93.6 |  |
|  | Ever Rural | 25 | 9.6 | 22 | 6.7 |  |  | 156 | 9.5 | 123 | 6.4 |  |
|  | Missing | ≤10 |  |  |  |  |  |  |  |  |  |  |
| Insurance | |  |  |  |  | **<0.001** |  |  |  |  |  | **<0.001** |
|  | Uninsured/No Record of Insurance | 59 | 22.6 | ≤10 |  |  |  | 712 | 43.4 | 17 | 0.9 |  |
|  | Public | 29 | 11.1 | 55 | 16.8 |  |  | 208 | 12.7 | 338 | 17.7 |  |
|  | Private | 166 | 63.6 | 262 | 80.1 |  |  | 662 | 40.4 | 1,429 | 74.8 |  |
|  | Other | ≤10 |  | ≤10 |  |  |  | 57 | 3.5 | 127 | 6.6 |  |
| Died during study | | ≤10 |  | ≤10 |  | 0.787 |  | 0 |  | 0 |  |  |
| Percentages are suppressed when count is ≤10  ^a^chi-squared or Fisher’s exact p-value: survivors vs population sample; p<0.05 bolded | | | | | | | | | | | | |

| Supplemental Table 2: Incidence Rate Ratios (IRRs), and 95% CIs for Concomitant HPV Vaccine Missed Opportunities by Vaccine Encounter Type by Demographic and Clinical Characteristics Among Pediatric Cancer Survivors | | | | | | | | | | |
| --- | --- | --- | --- | --- | --- | --- | --- | --- | --- | --- |
|  |  | Any Missed Opportunity | | | Other Adolescent Schedule | | | Flu Shot | | |
|  |  | IRR | 95% CI | p for Trend | IRR | 95% CI | p for Trend | IRR | 95% CI | p for Trend |
| Sex ^a^ | |  |  |  |  |  |  |  |  |  |
|  | Female | ref |  |  | ref |  |  | ref |  |  |
|  | Male | 1.12 | 1.00-1.25 |  | 1.33 | 0.97-1.83 |  | 1.00 | 0.95-1.05 |  |
| Race/Ethnicity ^a^ | |  |  |  |  |  |  |  |  |  |
|  | Other Race/Ethnicity | ref |  |  | ref |  |  | ref |  |  |
|  | Non-Hispanic White | 0.97 | 0.80-1.11 |  | 0.88 | 0.57-1.35 |  | 1.00 | 0.93-1.07 |  |
| Parental Education ^a^ | |  |  | **<0.001** |  |  | **0.012** |  |  | **0.016** |
|  | <High School | ref |  |  | ref |  |  | ref |  |  |
|  | High School/GED | **1.43** | **1.17-1.74** |  | **2.39** | **1.35-4.22** |  | **1.21** | **1.07-1.38** |  |
|  | Some College/AA | **1.54** | **1.26-1.88** |  | **2.67** | **1.53-4.64** |  | **1.20** | **1.05-1.38** |  |
|  | ≥College | **1.53** | **1.24-1.88** |  | **2.42** | **1.29-4.56** |  | **1.22** | **1.06-1.40** |  |
| Rural/Urban ^a^ | |  |  |  |  |  |  |  |  |  |
|  | Urban | ref |  |  | ref |  |  | ref |  |  |
|  | Ever Rural | **1.27** | **1.09-1.49** |  | 1.71 | 0.84-3.46 |  | 1.05 | 0.99-1.10 |  |
| Insurance ^a^ | |  |  |  |  |  |  |  |  |  |
|  | Uninsured/No Record of Insurance | 0.89 | 0.52-1.55 |  | --- | --- |  | 1.06 | 1.00-1.13 |  |
|  | Public | 0.98 | 0.82-1.17 |  | 0.90 | 0.57-1.41 |  | 1.00 | 0.90-1.11 |  |
|  | Private | ref |  |  | ref |  |  | ref |  |  |
|  | Other | 0.96 | 0.84-1.10 |  | 0.74 | 0.33-1.67 |  | 0.95 | 0.81-1.12 |  |
| Age at First Vaccine ^a^ | |  |  | **<0.001** |  |  | 0.531 |  |  | **0.012** |
|  | 9-10 | **1.38** | **1.20-1.58** |  | 1.20 | 0.87-1.66 |  | **1.09** | **1.02-1.16** |  |
|  | 11-12 | ref |  |  | ref |  |  | ref |  |  |
|  | 13-16 | 1.16 | 0.92-1.45 |  | **1.73** | **1.19-2.54** |  | 1.02 | 0.89-1.16 |  |
| ICCC Diagnosis Group ^b^ | |  |  |  |  |  |  |  |  |  |
|  | Leukemia | 1.06 | 0.74-1.53 |  | 0.85 | 0.34-2.13 |  | 1.05 | 0.77-1.43 |  |
|  | Lymphoma | 0.94 | 0.64-1.39 |  | 0.69 | 0.25-1.87 |  | 1.01 | 0.74-1.36 |  |
|  | CNS | 1.17 | 0.83-1.64 |  | 1.08 | 0.43-2.73 |  | 1.15 | 0.87-1.52 |  |
|  | Solid Tumors | 1.06 | 0.75-1.49 |  | 0.81 | 0.33-2.02 |  | 1.06 | 0.79-1.42 |  |
|  | Sarcomas/Bone | 1.25 | 0.87-1.80 |  | 1.51 | 0.60-3.79 |  | 1.09 | 0.81-1.47 |  |
|  | Epithelial | ref |  |  | ref |  |  | ref |  |  |
| Diagnosis Age ^b^ | |  |  | **<0.001** |  |  | 0.085 |  |  | 0.220 |
|  | 0-4 | ref |  |  | ref |  |  | ref |  |  |
|  | 5-9 | **1.21** | **1.08-1.35** |  | 1.20 | 0.86-1.69 |  | 1.05 | 1.00-1.11 |  |
| Treatment ^b^ | |  |  |  |  |  |  |  |  |  |
|  | No Chemotherapy | ref |  |  | ref |  |  | ref |  |  |
|  | Chemotherapy | **1.35** | **1.12-1.62** |  | **1.48** | **1.01-2.17** |  | **1.15** | **1.00-1.32** |  |
|  |  |  |  |  |  |  |  |  |  |  |
|  | No Radiation | ref |  |  | ref |  |  | ref |  |  |
|  | Radiation | 0.88 | 0.76-1.03 |  | 0.73 | 0.47-1.14 |  | **0.92** | **0.85-0.99** |  |
| ^a^ Models adjusted for all demographic factors simultaneously; cells left blank when there were insufficient events for estimation; p<0.05 bolded  ^b^ Models were adjusted for sex, race/ethnicity, and age at first vaccine in addition to clinical risk factors cells left blank when there were insufficient events for estimation; p<0.05 bolded | | | | | | | | | | |
